# Refining the Assessment of Entitlement in Romantic Relationships: The Sense of Relational Entitlement Scale—Revised (SRE-R)

**DOI:** 10.3389/fpsyg.2021.744618

**Published:** 2021-09-27

**Authors:** Rami Tolmacz, Lilac Lev-Ari, Rachel Bachner-Melman

**Affiliations:** ^1^Interdisciplinary Center Herzliya, Herzliya, Israel; ^2^Ruppin Academic Center, Emek Hefer, Israel; ^3^The Lior Tsfaty Center for Suicide and Mental Pain Studies, Ruppin Academic Center, Emek Hefer, Israel; ^4^Hebrew University of Jerusalem, Jerusalem, Israel

**Keywords:** Sense of Relational Entitlement scale, attachment, pathological concern in relationships, authenticity in relationships, romantic relationships

## Abstract

**Objective:** A subjective sense of entitlement is strongly evoked in the context of romantic relationships. A pathological sense of entitlement results from believing a partner should fulfill all one’s needs and wishes (inflated) or that the expression of genuine needs is illegitimate (restricted). This study aimed to validate a revised, improved version of the Sense of Relational Entitlement scale entitled the Sense of Relational Entitlement scale—Revised (SRE-R). We hypothesized it would have good factor structure and convergent validity, and that attachment dimensions and relationship indices would predict both inflated and restricted subscales.

**Method:** The SRE-R was completed by 854 Israeli adults (8.3% males) aged 31.94 ± 8.02. A subset (*n* = 629) also completed measures of attachment (ECR-S) and 447 completed measures of relational authenticity, pathological concern, and relational obsessions and compulsions.

**Results:** CFA confirmed two factors, inflated and restricted sense of relational entitlement. Relational obsessive-compulsive symptoms and pathological concern predicted an inflated sense of entitlement, and attachment avoidance, pathological concern, and authenticity in relationships predicted a restricted sense of entitlement.

**Discussion:** The SRE-R is a valid and useful tool to assess sense of entitlement in romantic relationships and should be examined in diverse samples and cultures.

## Introduction

Our sense of relational entitlement, or the subjective perception of what we deserve in a specific relational situation, influences our interactions, and attitudes in a wide range of contexts (Tolmacz, 2011). Entitlement is as old as society itself but has received increasing attention in psychological discourse and research over the past few decades. For example, Samuelson (1995) described an “age of entitlement” in which Americans believe that almost everyone deserves to succeed, and Twenge (2014), referred to the children of baby boomers born 30–40 years ago as “The Entitlement Generation.”

Traditionally, a sense of relational entitlement has been perceived in pathological terms, for example as a symptom of narcissistic personality disorder (American Psychiatric Association, 2000) or criterion for psychopathy (Hare, 1999). However, some researchers have expanded the concept to include the healthy assertion of needs and rights (Levin, 1970; Kriegman, 1983; Moses and Moses-Hrushovski, 1990). Wolfe and Bailey (2003) and Tolmacz (2011) have reconceptualized a sense of relational entitlement, based on the developmental perspective of attachment theory. In their understanding, a sense of entitlement develops largely in the context of attachment relationships with primary caregivers. Different sense of entitlement patterns are formed within an individual’s “internal working models” or mental representations of the self and relationship partners (Bowlby, 1973). This perspective frames sense of relational entitlement as a universal phenomenon manifested throughout the lifetime and encompassing both pathological and healthy assertion of needs and rights, rather than as an unequivocal expression of psychopathology.

In adulthood, romantic partners become central attachment figures, and maintaining proximity to them in times of need becomes a crucial source of support, comfort, and reassurance (Mikulincer, 2006). Extensive research has documented the central role of a person’s dominant attachment orientation to motives, cognitions, feelings, and behavior in romantic relationships (see Feeney, 2016, for review). Thus, the conceptualization of sense of entitlement in terms of attachment theory suggests that although one’s general sense of entitlement can affect attitudes and behavior in a wide range of relational contexts (Kriegman, 1983; Moses and Moses-Hrushovski, 1990), it has unique relevance and importance in the context of romantic relationships. These dyadic relationships seem to generate wants, needs, and expectations of the kind we would not expect to find in other relationships, rendering this intersubjective field particularly fertile for the study of entitlement patterns. This presumption is also supported by clinical evidence (Tolmacz, 2011). For this reason, Tolmacz and Mikulincer (2011) developed and validated the Sense of Relational Entitlement scale (SRE), which measures subjective expectations for the fulfillment of relational needs and wishes by a romantic partner, and cognitive and affective responses to a partner’s failure to meet them.

The original SRE scale contains 33 items organized around three basic entitlement-related attitudes toward a romantic partner: an assertive sense of entitlement, an inflated sense of entitlement, and a restricted sense of entitlement. An assertive sense of entitlement reflects a confident sense of self in romantic relationships and a reluctance to subjugate one’s own needs to those of one’s partner. People with an assertive sense of entitlement are able to recognize both their own and their partners’ subjectivities, without one negating the other (Tolmacz, 2011). Individuals with an inflated sense of entitlement expect their partners to take care of their needs and wishes, are highly sensitive and responsive to violations of such expectations, and experience regret about their relationships and their partners. Finally, individuals with a restricted sense of entitlement are characterized by a lack of assertion and inhibited expression of their needs and expectations in romantic relationships and perceived lack of worthiness in their partners’ eyes. They seem to doubt their right to express genuine wishes and needs. Research has shown associations between both styles of imbalanced sense of entitlement (restricted and inflated) and attachment insecurities, emotional difficulties, low levels of well-being, dissatisfaction with romantic relationships (Tolmacz and Mikulincer, 2011; George-Levi et al., 2014; Gerber et al., 2015) and pathological concern, a relational attitude characterized by repression and denial of self-related needs and overinvestment in satisfying others’ needs.

Clearly, the SRE scale has enriched our understanding of the role played by sense of entitlement in romantic relations. However, there are some drawbacks to its use. First, while 23 items measure an inflated sense of entitlement, only six measure assertive entitlement and four measure restricted entitlement. Moreover, assertive entitlement, as assessed by the SRE scale, has consistently failed to show significant positive associations with measures of psychological functioning and adjustment, such as positive mood, life satisfaction, and marital adjustment, and significant negative associations with measures of emotional problems and relational distress (e.g., Tolmacz and Mikulincer, 2011; Chakir and Feldman, 2020; Leiman, 2020). This has led Tolmacz and Mikulincer (2011) to conclude that “we might have failed to assess the specific facets of functioning and adjustment that assertive entitlement can contribute, such as assertiveness, social power, or control. Further studies should further examine the psychological correlates and outcomes of a sense of assertive entitlement in close relationships” (p. 91). Moreover, a closer examination of the items intended to measure an assertive sense of entitlement suggests that they are likely to be endorsed also by individuals with an inflated sense of entitlement. These items are: “I won’t make do with less than what I deserve in a couple relationship” (item 7); “I insist on getting what I deserve in my relationship” (item 30); “I am unable to make compromises in choosing a partner” (item 19); “I think my partner is lucky to be with me” (item 28); “I deserve to get in my relationship things I was deprived in prior relationships” (item 25), and “I deserve a partner who is very sensitive” (item 18). Two recent studies by Candel and Turliuc (2019, 2021) failed to find a consistent pattern of associations between assertive sense of relational entitlement and other relational indices, supporting Tolmacz and Mikulincer (2011) concern about the assertive subscale.

In further validation of the SRE by George-Levi et al. (2014), the intercorrelations between “assertive entitlement” and “entitlement expectations” (one of the factors that in the original study (2011) seemed to tap different aspects of inflated forms of entitlement) were found to be significant and positive. The authors suggested that these two factors representing a more secure, mature, healthy, or assertive sense of entitlement and suggested that the entitlement expectations items should not be included in the inflated factor. Following their recommendation, 7 items of the inflated factor were not included in the two remaining factors that seem to tap different aspects of inflated forms of entitlement: (a) vigilance regarding negative aspects of partner and relationship, and (b) sensitivity to relational transgressions and frustrations. Another item “When my partner makes me angry, I sometimes regret the fact that I don’t have a different partner” was adapted from the inflated factor on the Sense of Relational Entitlement of Adolescents Toward their Parents (SRE-ap) (Tolmacz et al., 2016). In addition, we added another three items tapping restricted sense of entitlement to the original restricted items: Item 8 –“Usually, when my partner compliments me, I believe I do not deserve the compliment”; Item 13-“Sometimes, I think my partner loves me more than I deserve.” And item 9- “I think my partner deserves someone more successful than me.” The restricted entitlement factor of the revised scale therefore includes seven items and the total SRE-R 15 items.

This 15-item Sense of Relational Entitlement—Revised scale (SRE-R), is examined in this study and it is hoped that it will overcome some of the shortcomings of the SRE scale. Sense of relational entitlement has been conceptualized in terms of attachment theory (Tolmacz, 2011) and the SRE-R adopts an approach to the measurement of sense of relational entitlement that is parallel to that of the Experiences in Close Relationships scale (ECR; Brennan et al., 1998), widely used to assess attachment style in romantic relationships. Just as the ECR taps two problematic dimensions of attachment in relationships (anxiety and avoidance), the SRE-R taps two problematic dimensions of entitlement (inflation and restriction). Low levels of both inflated and restricted entitlement as assessed using the SRE-R are theorized to indicate a healthy, or assertive sense of entitlement in romantic relationships, in the same way as low levels of attachment anxiety and avoidance are indicative of secure attachment.

We administered the SRE-R to a large community sample of adults in significant romantic relationships and aimed to validate its two-factor structure (inflated entitlement, restricted entitlement). We also aimed to replicate associations found in previous studies (that used the 33-item SRE scale) between sense of relational entitlement and other relational variables, such as attachment orientations and pathological concern (e.g., Tolmacz and Mikulincer, 2011; George-Levi et al., 2014; Shavit and Tolmacz, 2014). In addition, we aimed to examine associations between the two SRE-R factors, relational obsessions/compulsions and authenticity in romantic relationships. Authenticity refers to behavior consistent with feelings, attitudes and beliefs and involves being genuine in connections with other people (Wood et al., 2008). Previous studies have indicated that within romantic relationships, lower levels of authenticity are associated with lower relational trust, commitment and satisfaction (Lopez and Rice, 2006; Gillath et al., 2010). Since restricted sense of relational entitlement is associated with low self-esteem (Tolmacz and Mikulincer, 2011), we hypothesized that it would be associated with relational inauthenticity.

Relationship obsessive compulsive disorder (ROCD) is characterized by obsessive–compulsive symptoms focusing on intimate relationships. It often involves preoccupations and doubts centered on one’s feelings toward a relationship partner, the partner’s feelings toward oneself, the “rightness” of the relationship experience (Doron et al., 2012b) and perceived flaws of one’s relationship partner (Doron et al., 2012a). Since both inflated and restricted entitlement both involve ongoing evaluation of the “worthiness” of self and partner, we hypothesized that both forms of imbalanced SRE-R would be positively associated with ROCD.

Whereas both inflated and restricted sense of entitlement have been shown to be significantly associated with attachment dimensions, intercorrelations between these variables in previous research have been weak to moderate (Tolmacz and Mikulincer, 2011; Tolmacz et al., 2016), so that attachment and entitlement dimensions are both connected and distinct. We therefore hypothesized not only that they would be significantly linked, but also that attachment would not fully explain the contribution of relationship indices (Roci, PCQ and AIRS) to inflated and restricted entitlement. In other words, we expected these relationship indices to predict inflated and restricted entitlement over and above attachment dimensions.

We hypothesized that:

1. The SRE-R will demonstrate good construct structure [using confirmatory factor analysis (CFA)].
2. The SRE-R will demonstrate good convergent validity, correlating positively with measures of attachment insecurities dimensions, pathological concern, and relational obsessions and compulsions. In addition, restricted entitlement will correlate and negatively with authenticity in romantic relationships.
3. Attachment dimensions will predict inflated and restricted sense of relational entitlement, and relationship indices (relational obsessions and compulsions, pathological concern, authenticity in relationships) will predict inflated and restricted sense of relational entitlement over and beyond attachment dimensions.

## Materials and Methods

### Participants

A total of 854 (8.3% males) participants between 18 and 61 years of age (*M* = 31.94, *SD* = 8.02) registered online to participate in the study and completed the SRE-R. All participants reported having been in significant romantic relationships at the time of the study. A subset of 629 participants also completed a short version of the ECR, and 447 completed three additional measures, of authenticity in relationships, pathological concern, and relational obsessions and compulsions. Community volunteers were recruited via social networks. Most participants were born in Israel (83.7%) and were single (71.5%). Almost half (47.01%) had graduate degrees and 46.32% had high school diplomas.

### Measures

#### Sense of Entitlement in Romantic Relationships

To assess sense of relational entitlement, the participants completed the 15-item SRE-R. Nine items were taken from the original SRE (Tolmacz and Mikulincer, 2011) and the other six were adapted from the SRE-ap (Tolmacz et al., 2016). The SRE-R has two subscales: 1. A seven-item restricted subscale that evaluates (a) the extent to which the respondent’s ability to express his/her wishes, expectations and needs in relationships is limited and inhibited and (b) perceived lack of deservingness in the partner’s eyes. Examples are “Usually, when my partner compliments me, I believe I do not deserve the compliment” and “I often feel my partner deserves someone better than me”; and 2. An eight-item inflated subscale that assesses (a) the extent to which the respondent’s expectations and demands from his/her romantic partner is excessive, and (b) feelings of regret about the current partner and relationship. Examples are “I spend a lot of time thinking about my partner’s weaknesses” and “When my partner makes me angry, I sometimes regret the fact that I don’t have a different partner.” Responses are scored on a five-point Likert type scale (1–5). The SRE-R was written in Hebrew by R.T. In this study the Cronbach’s α for the inflated entitlement subscale was 0.85 and for the restricted entitlement subscale 0.91.

### Attachment Dimensions

Anxious and avoidant attachment were assessed with the Emotions in Close Relationships Scale—Short Form (ECR-S, Wei et al., 2007). Participants were asked to what extent they agree with statements about interpersonal relationships and connections. The ECR-S is a 12-item questionnaire with two subscales, a six-item anxiety subscale and a six-item avoidance subscale. Responses are marked on a seven-point Likert type scale (1–7). An example of an item from the anxiety subscale is “I worry a lot about my relationships,” and an example of an item from the avoidance subscales is “I tell my close relationship partners just about everything (reversed).” Mean subscale scores reflect levels of anxiety and avoidance. The ECR-S has good validity, reliability and test-retest reliability over 3 weeks (Wei et al., 2007). In this study the Cronbach’s α for the anxiety subscale was 0.85 and for the avoidant subscale was 0.86.

### Relationship Indices

#### Pathological Concern

Pathological concern was measured using the Pathological Concern Questionnaire (PCQ; Shavit and Tolmacz, 2014). The PCQ contains 18 items and inquires about thoughts, feelings and behaviors related to two aspects of pathological concern: (1) repression and denial of one’s own needs; and (2) excessive investment in satisfying the needs of others. Responses are scored on a seven-point Likert type scale (1–7), with higher scores indicating greater pathological concern. The original questionnaire was written in Hebrew and found to be reliable and valid (Shavit and Tolmacz, 2014). In this study the Cronbach’s α was 0.90.

#### Authenticity in Romantic Relationships

The degree of authenticity in romantic relationships was assessed using the Authenticity in Relationships Scale (AIRS; Lopez and Rice, 2006). This scale contains 21 items and asks about thoughts and feelings related to two facets of authenticity in relationships: (1) Intimate Risk Taking, or openness and personal disclosure to one’s partner; and (2) Unacceptability of Deception, or objection to an unauthentic stance taken by oneself or one’s partner. Items are scored on a 9-point Likert type scale (1–9), with higher scores indicating greater authenticity in romantic relationships. The AIRS has been found to be reliable and valid (Lopez and Rice, 2006). In this study the Hebrew translation used (Paldi, 2020) yielded a Cronbach’s α of 0.91.

#### Relational Obsessions and Compulsions

Obsessions and compulsions centered on one’s romantic relationship were measured by the Relationship Obsessive-Compulsive Inventory (ROCI; Doron et al., 2012b). The ROCI is a 12-item self-report scale assessing three relational dimensions: (1) feelings toward one’s partner (e.g., “I continuously doubt my love for my partner”); (2) partner’s feelings toward oneself (e.g., “I keep asking my partner whether she/he really loves me”); and (3) the rightness of the relationship (e.g., “I check and recheck whether my relationship feels right”). Participants rank their endorsement of these statements on a five-point Likert type scale (0–4). Total and subscale scores correlate significantly with measures of stress, relationship quality and symptoms of OCD, anxiety and depression (Doron et al., 2012a). The internal consistencies of the subscales in our sample (Cronbach’s alphas) ranged from 0.84 to 0.89. The internal consistency of the entire scale was 0.93.

#### Procedure

The study received approval from the Institutional Internal Review Board. Volunteer participants were recruited via the social media and via psychology B.A. courses in exchange for class credit. When participants opened the link sent to them, a full explanation about the study appeared on the first screen, and they were asked to provide informed consent. After reporting on demographic information, they completed the SRE-R, PCQ, AIRS, and ROCI. On the last screen, contact details of the researchers were provided and participants were encouraged to reach out to them with questions, comments or difficulties.

#### Data Analyses

No relations between demographic variables and SRE were found. AMOS 23.0 was used to conduct the CFA. To test for convergent validity, Pearson correlations were calculated between SRE-R subscales scores and relationship indices (PCQ, AIRS, and ROCI). Hierarchical regression analyses were assessed in order to predict SRE from attachment and relationship indices. Analyses were conducted using the Statistical Package for the Social Sciences (SPSS, version 23).

## Results

Hypothesis 1: *The SRE-R will demonstrate good construct structure (using CFA).*

### CFA for the SRE-R (*N* = 854)

**FIGURE 1:**
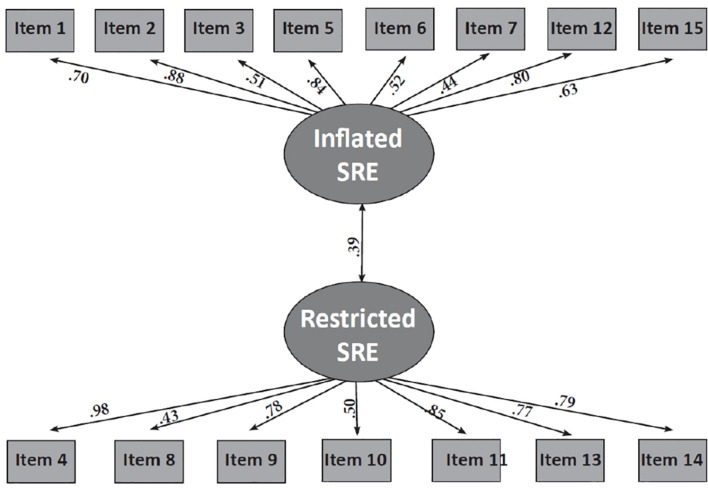
CFA of the two-factor model for the SRE-R. All paths were statistically significant at *p* < 0.001. Latent variables are indicated by ellipses. Observed variables are indicated by rectangles. Arrows between latent variables signify correlations between latent variables. Correlations between latent and observed variables were significant at *p* < 0.001.

CFA examines whether the data is consistent with the constructs (factors) as hypothesized theoretically or empirically, in this case inflated and restricted sense of relational entitlement. The following values were chosen as conditions for acceptance of the model: Comparative Fit Index (CFI) > 0.90 (Bentler and Bonett, 1980), root mean square error of approximation (RMSEA) < 0.08 (Browne and Cudeck, 1993), and SRMR < 0.08 (see Figure 1 and **Appendix A**). The model showed good fit for the data [χ^2^_(__59__)_ = 144.35; *p* < 0.001; CFI = 0.99, RMSEA = 0.04; SRMR = 0.02]. Cronbach’s alphas were 0.85 for inflated entitlement and 0.91 for restricted sense of entitlement.

Hypothesis 2: *The SRE-R will have good convergent validity: It will correlate positively with measures of attachment dimensions (ECR-S), pathological concern (PCQ), relational obsessions and compulsions (ROCI), and negatively with authenticity in relationships (AIRS).*

Pearson correlations between the SRE-R subscale scores and ROCI, PCQ, AIRS and ECR-S subscale scores are presented in Table 1. All correlations were significant at *p* < 0.001 and ranged between 0.21 and 0.66.

Hypothesis 3: *Attachment dimensions (ECR-S avoidance and anxiety) will predict inflated and restricted SRE, and relationship indices (ROCI, PCQ, AIRS) will predict inflated and restricted SRE over and beyond attachment dimensions.*

**TABLE 1:** Correlations between SRE-R subscales and ROCI, PCQ, AIRS, and ECR-S subscale scores (min *n* = 446).

	Inflated SRE	Restricted SRE	ROCI	ECR-S anxiety	ECR-S avoidance	PCQ	Mean (*SD*)
Inflated SRE							23.6 (0.83)
Restricted SRE	0.36						1.75 (0.79)
ROCI	0.53	0.22					1.67 (0.99)
ECR-S anxiety	0.39	0.32	0.31				3.39 (1.23)
ECR-S avoidance	0.21	0.21	0.33	0.19			3.24 (1.07)
PCQ	0.66	0.39	0.65	0.55	0.36		2.79 (0.98)
AIRS	−0.46	−0.37	−0.55	−0.29	−0.45	−0.57	5.69 (0.83)

*All correlations were significant at the p < 0.001 (2-tailed).*

*SRE, Sense of Relational Entitlement; ROCI, Relationship Obsessive-Compulsive Inventory; ECR-S, Experiences in Close Relationships Scale—Short Form; PCQ, Pathological Concern Questionnaire; AIRS, Authenticity in Relationships Scale.*

To assess whether ECR-S avoidance and anxiety would predict inflated and restricted SRE-R, and whether relational indices (ROCI, PCQ, AIRS) would predict inflated and restricted SRE-R over and beyond ECR-S avoidance and anxiety, we performed two hierarchical regression analyses (see Table 2). Inflated and restricted sense of entitlement were the dependent variables. In the first step, attachment dimensions were entered into the model and in the second step, ROCI, AIRS, and PCQ were added.

**TABLE 2:** Prediction of inflated and restricted SRE using ECR-S anxious and avoidant attachment, ROCI, AIRS and PCQ (*n* = 460).

Predictor	Inflated SRE	Restricted SRE
Step 1		
R^2^/Adj. R^2/^F_(_*_*df*_*_)_	0.22/0.21/_(__5_,_262__)_ = 36.78***	0.15/0.15/_(__5_,_262__)_ = 23.86***
ECR-S anxiety	0.40***	0.27***
ECR-S avoidance	0.18**	0.24***
Step 2		
R^2^/Adj. R^2/^F_(_*_*df*_*_)_/ΔR	0.52/0.51/0.30***/_(__5_,_259__)_ = 54.92***	0.23/0.21/0.30***/_(__5_,_259__)_ = 15.14***
ECR-S anxiety	0.08	0.12
ECR-S avoidance	0.003	0.12*
ROCI	0.32***	0.03
AIRS	−0.07	−0.15*
PCQ	0.37***	0.21*

*p < 0.1, **p < 0.05, ***p < 0.01.

*Numbers depict standardized β.*

*ECR-S, Experiences in Close Relationships scale—Short Form; SRE, Sense of Relational Entitlement; ROCI, Relationship Obsessive-Compulsive Inventory; AIRS, Authenticity in Relationships Scale; PCQ, Pathological Concern Questionnaire.*

As can be seen from Table 2, **ECR-S anxious and avoidant** attachment dimensions initially predicted inflated and restricted SRE, explaining 22 and 15% of the variance, respectively. For inflated SRE, entering other relationship indices into the model eliminated the predictive value of the ECR-S attachment dimensions. ROCI and PCQ then became positive predictors of inflated SRE, increasing the explained variance to 51%. For restricted SRE, entering other relationship indices into the model eliminated the predictive value only of ECR-R attachment anxiety. ECR-S attachment avoidance and PCQ were positive predictors and AIRS was a negative predictor of SRE, increasing the explained variance to 21%.

## Discussion

Clinical evidence and theoretical writings point to the importance and uniqueness of the sense of entitlement in couple relationships. Individuals high in entitlement and involved in romantic relationships display a pattern of selfishness (Campbell et al., 2004) and have trouble forgiving their partners (Exline et al., 2004). Men high on entitlement were also found to be characterized by a sense of propriety, which can lead to violent behavior toward female partners (Hannawa et al., 2006). This line of research led Tolmacz and Mikulincer (2011) to develop and validate the Sense of Relational Entitlement (SRE) scale, to assess individual differences in sense of entitlement in romantic relationships. The scale has frequently been used in psychological research, particularly in context of problematic relationships (e.g., George-Levi et al., 2014; Brenner et al., 2019; Efrati et al., 2019). However, a disproportionate number of SRE items assessing an inflated sense of entitlement (23 out of 33). In addition, the assertive subscale failed to correlate with similar indices conceptually related to well-being and relationship satisfaction. The purpose of this study was therefore to validate the Hebrew version of the Sense of Relational Entitlement—Revised scale (SRE-R). Since the SRE conceptualized a sense of relational entitlement in terms of attachment theory (Tolmacz, 2011), the rationale behind the structure of the ECR scale was adopted. The SRE-R therefore assesses two pathological dimensions of entitlement (inflation and restriction), and healthy entitlement is operationalized as low scores on these dimensions.

Using CFA, a two-factor model was supported and the factor structure of the Hebrew version of the SRE-R was confirmed. The Inflated and Restrictive Entitlement subscales demonstrated good internal reliability and positive, moderate intercorrelations.

The SRE-R also showed good convergent validity. Participants who reported higher levels of Inflated or Restrictive entitlement also scored higher in anxious attachment, avoidant attachment, obsessive-compulsive relationship behaviors and pathological concern in relationships and lower in relational authenticity. This information supports the construct validity of the questionnaire.

The fact that insecure attachment orientations (anxious and avoidant) both initially predicted imbalanced type of sense of entitlement (inflated and restricted) corroborates previous research findings (e.g., Tolmacz and Mikulincer, 2011; Brenner et al., 2019). More specifically, Tolmacz and Mikulincer (2011) found a positive association between inflated sense of entitlement and anxious attachment, so that inflated sense of relational entitlement seems to involve a hyperactivation of attachment wishes, needs and fears (Mikulincer and Shaver, 2007). The authors concluded that “inflated entitlement demands may reflect the same frustration and dissatisfaction with relationship partners that lead to anxious attachment. As such, the partial and unsystematic recognition of one’s entitlement by others may be one particular aspect of close relationships that is internalized into negative working models of the self and contribute to both anxious attachment and inflated entitlement in romantic relationships” (p. 91).

It also ties in with the conceptualization of sense of relational entitlement as an aspect of internal working models according to attachment theory. Moreover, it supports the perception that an imbalanced sense of relational entitlement can stem, at least in part, from frustrating interactions with primary caregivers (Tolmacz, 2011). When other relationship indices were entered into the model predicting sense of entitlement, both pathological concern and obsessive-compulsive traits in relationships, but not attachment, were significant predictors of an inflated sense of relational entitlement. It seems intuitive that this type of entitlement, focused on the belief that one deserves to have their needs and wishes fulfilled by a partner, should be associated with relational obsessive-compulsive tendencies. These tendencies predispose people to focus on preoccupations and doubts about the “rightness” of the relationship experience (Doron et al., 2012b) and about the perceived flaws of one’s partner (Doron et al., 2012a). Moreover, since an inflated sense of entitlement characterizes imbalanced relationships, a strong association with other characteristics of imbalanced relationships such as pathological concern is not surprising. Pathological concern and relationship obsessive-compulsive behaviors may be two maladaptive strategies linking insecure attachment orientations with an inflated sense of entitlement in romantic relationships.

When relationship indices were entered into the model predicting a restricted sense of relational entitlement, avoidant attachment and pathological concern were positive predictors and relational authenticity a negative predictor. For individuals with a restricted sense of relational entitlement, being authentic in intimate relationships might be perceived as a risk because of shame, and/or as an undeserved privilege since genuine needs are seen as illegitimate (Blavier and Glenn, 1995). In accordance with Gabbard (1989) claim that “hypervigilant narcissists” in fact continually direct their attention toward others, pathological concern may be a way of avoiding authenticity and allowing others to enjoy the expression of real needs denied to self. Both pathological concern and inauthenticity may be misguided strategies to cope with frustrating attachment relationships and a strong negative association has indeed been observed between pathological concern and authenticity (Tolmacz et al., 2021a, b).

Our study has several limitations. First, the SRE-R used in this study was written in Hebrew, so its psychometric properties should be examined in other languages. Second, participants were predominantly female and highly educated, so results may not be generalizable to other populations. Future studies should investigate the validity of SRE-R in samples of more diverse genders, socioeconomic and cultural backgrounds and ethnicities. Third, we did not ask about sexual orientation in this study, so the connection of sense of entitlement and sexual orientation should be examined. Finally, participants were not asked about the duration of their relationship, which may be connected to a sense of relational entitlement. Future studies should examine relationships of diverse durations and include partners in long-term relationships. The SRE-R could be administered using a dyadic paradigm to examine variables, such as emotional regulation, that may mitigate the negative impact of entitlement on relationship outcomes.

Because of the limitations of the original SRE, the goal of the current study was to validate a shorter, two-dimensional scale (SRE-R) to measure individual differences in sense of entitlement in the context of romantic relationships. We propose the SRE-R as a shorter, more user-friendly and parsimonious questionnaire and recommend its use in clinical and research settings around the globe.

## Data Availability Statement

The original contributions presented in the study are included in the article/supplementary material, further inquiries can be directed to the corresponding author/s.

## Ethics Statement

The studies involving human participants were reviewed and approved by the Ruppin Academic Center Ethics Committee. The patients/participants provided their written informed consent to participate in this study.

## Author Contributions

RT conceived and planned the study, adapted the questionnaire, oversaw recruitment, and co-authored the manuscript. LL-A performed all statistical analyses and wrote sections “Materials and Methods” and “Results.” RB-M helped the co-author in the writing and editing of the manuscript.

## Conflict of Interest

The authors declare that the research was conducted in the absence of any commercial or financial relationships that could be construed as a potential conflict of interest.

## Publisher’s Note

All claims expressed in this article are solely those of the authors and do not necessarily represent those of their affiliated organizations, or those of the publisher, the editors and the reviewers. Any product that may be evaluated in this article, or claim that may be made by its manufacturer, is not guaranteed or endorsed by the publisher.

## Appendix

### SRE-R

The following statements concern attitudes, feelings, beliefs, and reactions in romantic relationships. Please respond to each statement by indicating how much you agree or disagree with it.

**Table:**

**1**	**2**	**3**	**4**	**5**
**Strongly disagree**	**Disagree**	**Neutral/mixed**	**Agree**	**Strongly agree**
	
1	When my partner makes me angry, I sometimes regret the fact that I don’t have a different partner.	1	2	3	4	5
2	I spend a lot of time thinking about my partner’s weaknesses.	1	2	3	4	5
3	When my partner hurts me, I’m immediately filled with a sense of distrust.	1	2	3	4	5
4	I often feel my partner deserves someone better than me.	1	2	3	4	5
5	Sometimes I have a lot of criticism toward my partner.	1	2	3	4	5
6	When my partner frustrates me, I get very angry.	1	2	3	4	5
7	When I am frustrated by my partner, I feel I do not deserve it.	1	2	3	4	5
8	Usually, when my partner compliments me, I believe I do not deserve the compliment.	1	2	3	4	5
9	I think my partner deserves someone more successful than me.	1	2	3	4	5
10	I often ask myself whether I deserve such a great partner.	1	2	3	4	5
11	I feel my partner deserves more than s/he gets from me.	1	2	3	4	5
12	When my partner frustrates me, I sometimes think of ending the relationship.	1	2	3	4	5
13	Sometimes, I think my partner loves me more than I deserve.	1	2	3	4	5
14	Sometimes I feel I’m not good enough for my partner.	1	2	3	4	5
15	Sometimes I’m angrier with my partner than with other people.	1	2	3	4	5

*Inflated sense of entitlement: 1, 2, 3, 5, 6, 7, 12, 15.*

*Restricted sense of entitlement: 4, 8, 9, 10, 11, 13, 14.*
